# Application of the Bioabsorbable Polyglycolic Acid Sheet in Colorectal Anastomosis in Animal Models

**DOI:** 10.14789/jmj.JMJ22-0001-OA

**Published:** 2022-08-15

**Authors:** SHINGO KAWANO, YUTAKA KOJIMA, YUKI TSUCHIYA, SHUNSUKE MOTEGI, RYOICHI TSUKAMOTO, KAZUMASA KURE, KIICHI SUGIMOTO, MAKOTO TAKAHASHI, ATSUSHI OKUZAWA, KAZUHIRO SAKAMOTO

**Affiliations:** 1 Department of Coloproctological Surgery, Juntendo University Factually of Medicine, Tokyo, Japan; 1 Department of Coloproctological Surgery, Juntendo University Factually of Medicine, Tokyo, Japan

**Keywords:** colorectal anastomosis, bioabsorbable polyglycolic acid sheet, animal model, laparoscopic anterior resection, double stapling technique

## Abstract

**Objectives:**

Anastomotic complications after colorectal surgery are one of the most serious outcomes. To address this issue, this study used the newly developed bioabsorbable polyglycolic acid (PGA) sheet to assess its usefulness and safety using two approaches of double stapling technique (DST) after laparoscopic anterior resection (AR) in pig models.

**Methods:**

Rectal intratissue pressure was assessed after DST anastomosis in two groups, i.e., with (PGA group) or without PGA sheet (nonPGA group), which was sandwiched between the anastomosis in the first approach. In the second approach, after laparoscopic DST anastomosis with PGA sheet attached at anvil side, the clinical short-term outcomes within 1 week and histological findings at 1 week after the surgery were evaluated.

**Results:**

Assessment of rectal intratissue pressure showed a mean pressure of 9.28 kPa in the PGA group versus 5.78 kPa in the nonPGA group (*p* = 0.39). The results of clinical short-term outcomes revealed that there were no anastomotic complications. The results of histological findings in anastomotic bowel tissues with PGA sheet were not significantly different from those of the control case.

**Conclusions:**

The bioabsorbable PGA sheet can be used for colorectal DST anastomosis in animal models and may be a valuable tool for this procedure.

## Introduction

Anastomotic complications after colorectal surgery are the main cause of postoperative morbidity, mortality, impaired quality of life, and prolonged hospital stay, in addition to creating the risk for permanent stoma. Anastomosis complications generally include leakage, bleeding, and stenosis^1, 2)^. Anastomotic leakage (AL) is one of the major complications of colorectal surgery, especially in patients with rectal cancer. The occurrence of AL varies widely from 3% to 30% and increases the postoperative mortality rate^3-9)^. An option that holds promise to control leakage is that of staple line reinforcement, although use of this reinforcement in general is not new, previously described for bariatric surgery^10, 11)^, pancreatic surgery^12)^, and colorectal surgery^13, 14)^.

Several previous studies have assessed bioabsorbable felt constituted from polyglycolic acid (PGA)/trimethylene carbonate. Franklin et al reported the first series of cases using staple line reinforcement in colon surgery, in which the bioabsorbable felt was loaded on a linear stapler^14)^. The initial study results were very promising, with no bleeding and no significant leakage in a short-term follow-up. More importantly, no adverse outcomes have been observed in almost 3 years of continuous use. Consten et al reported early results with this approach that showed a decreased incidence of hemorrhage and leakage after gastric surgery^10)^. Nguyen et al demonstrated that the use of bioabsorbable staple line reinforcement is safe and effective in the prevention of intraoperative staple line bleeding and postoperative gastrointestinal hemorrhage^11)^. Several authors agree that the use of bioabsorbable felt as staple line reinforcement seems to be safe and may be useful in preventing AL, bleeding, and, potentially, intraluminal stenosis as well. The use of bioabsorbable staple line reinforcement may play an important role in high-risk patients undergoing colorectal surgery, which include those who use steroids, have a longer intraoperative time, or have cancer, immunosuppression, and contamination and other issues. However, Placer et al reported that bioabsorbable staple line reinforcement in colorectal surgery does not reduce the rate of pooled anastomotic complications^15)^.

A new, thinner (0.15 mm), reinforcement material constituted only from PGA (NEOVEIL; Gunze, Osaka, Japan) has been developed. This new type of sheet with only PGA allows a shorter absorption time, and the influence for tissue is less from this sheet. This study assessed the usefulness and safety of this new PGA sheet using the colorectal double stapling technique (DST) anastomosis during laparoscopic anterior resection (AR) via two approaches in animal models. Approach A involved the evaluation of usefulness, made by assessing the rectal intratissue pressure (RIP) after DST anastomosis. Approach B involved the evaluation of safety, made by assessing the clinical short-term outcomes of DST anastomosis with PGA sheet and evaluating histological findings

## Materials and Methods

### Ethics Statement

The miniature pigs were purchased from ZEN- NOH Nagano. The animal studies were approved by the Animal Review Board of Juntendo University (Approval number: 1323).

### Surgical procedure

All procedures were performed under sterile conditions by surgeons responsible for the assigned procedure. The pigs were administered isoflurane via inhalation for anesthesia. A laparoscopic AR was performed with colorectal end-to-end anastomosis with DST. The resected colon was the approximately 5 cm. The anal side of rectum was cut using the Endo GIA Reinforced Reload with Tri-Staple Technology^TM^ or Endo GIA Reload with Tri-Staple^TM^ (Medtronic, Minneapolis, USA). The anvil was extracorporeally set into the oral side of the sigmoid colon. The colorectal anastomosis was intracorporeally performed using the EEA Circular Stapler with DST Series Technology^TM^ (Medtronic, Minneapolis, USA).

### Approach A

**Figure 1:**
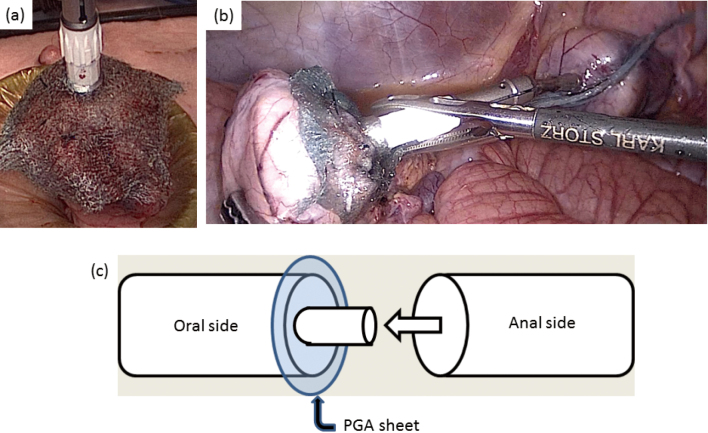
Colorectal anastomosis in the PGA group (Approach A) (a) The oral side of colon is covered with the PGA sheet outside of the abdominal space. (b) The PGA sheet is sandwiched between the oral side of the colon and the anal side of the rectum in anastomosis. (c) The schema of anastomosis in the PGA group.

**Figure 2:**
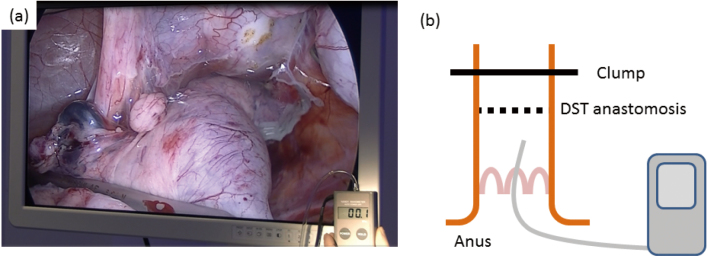
The schema of the measurement of RIP (a) The measurement of RIP was performed using HANDY MANOMETER PG-100 102GP. (b) The schema of the measurement of RIP. A sensor in the rectum was used for pressure measurement. The colon was clumped on the oral side of the anastomosis.

The animals, i.e., nine pigs were classified into two groups. The first was the PGA group. In this group, the anal side of rectum was cut using the Endo GIA Reinforced Reload with Tri-Staple Technology. The PGA sheet was used, which was sandwiched between the oral side of the colon and the anal side of the rectum in anastomosis (Figure 1). The second group was the nonPGA group. In this group, the anal side of rectum was cut using Endo GIA Reload with Tri-Staple. The PGA sheet was not used and was not sandwiched between the oral side of the colon and the anal side of the rectum in anastomosis. The measurement of RIP was performed using HANDY MANOMETER PG-100 102GP^TM^ (Nidec Copal Electronics, Tokyo, Japan) in the rectum, intraluminally filling it to the point of just overflowing by injecting air after anastomosis and clamping the oral side of the colon (Figure 2).

### Approach B

**Figure 3:**
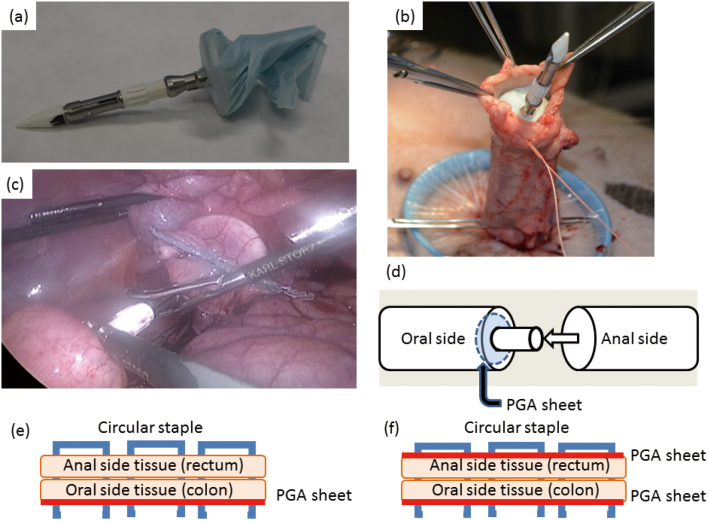
Colorectal anastomosis in the PGA group (Approach B) (a) The anvil was covered with the PGA sheet. (b) This anvil was set into the oral side of colon. (c) The PGA sheet was on the oral side of the colon with the anvil. (d) The schema of anastomosis. (e) The schema of staple and PGA sheet in this approach. One PGA sheet was used on the oral side. (f) The schema of staple and PGA sheet to be used in the future. Two PGA sheets were used on the oral and the anal side.

In this approach, laparoscopic AR was performed in four pigs. Rectal transection was performed using Endo GIA Reinforced Reload with Tri-Staple Technology in all pigs. In the first case, normal DST anastomosis was performed without using the PGA sheet. In the other three cases, the PGA sheet was extracorporeally attached to the anvil; subsequently, reinforced DST was performed (Figure 3(a)-(e)). After laparoscopic AR with DST, the clinical courses were observed for 1 week. Furthermore, second-look surgery was performed to observe the intracorporeal findings 1 week after the first operation, and anastomoses were resected and assessed histologically with hematoxylin and eosin stain. Anastomotic factors were clinically and histologically assessed.

### Statistical analysis

Comparison between the RIP of the PGA group and the nonPGA group was performed using Mann–Whitney U test. This analysis was performed using the JMP software (version 12.0, SAS Institute). All calculated *p* values were two-sided and *p* < 0.05 was considered statistically significant.

## Results

### Approach A: Measurement of RIP

**Figure 4:**
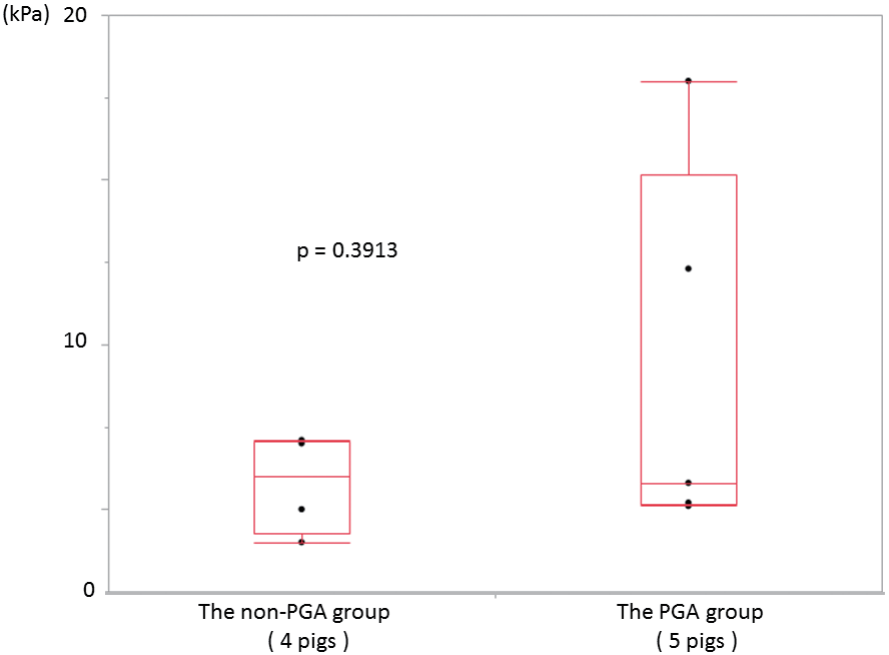
Measurement of RIP The mean RIP was 9.28 kPa for the PGA group versus 5.78 kPa for the nonPGA group, which did not reach statistical significance (*p* = 0.3913).

There were five pigs in the PGA group and four in the nonPGA group. The mean RIP was 9.28 kPa for the PGA group versus 5.78 kPa for the nonPGA group, which did not differ statistically (*p* = 0.39) (Figure 4).

### Approach B: Clinical Short-Term Outcomes

All four pigs were alive and had no event of clinical findings, such as fever, infection of incisional wound, or appetite loss, after undergoing laparoscopic AL with DST anastomosis. Although little postoperative adhesion was expected around the anastomosis during the second-look surgery, no anastomotic complications were evident in the abdominal cavity, such as bleeding, stricture formation, or leakage (Table 1).

**Table 1:** Result of Approach B

	PGA	Clinical complications of anastomosis			Histological findings in anastomotic tissue		
		leakage	bleeding	structure	leakage	Necrotic tissue	Abscess formation
Control case	(-)	(-)	(-)	(-)	(-)	(-)	(-)
Case 1	(+)	(-)	(-)	(-)	(-)	(-)	(+)
Case 2	(+)	(-)	(-)	(-)	(-)	(-)	(-)
Case 3	(+)	(-)	(-)	(-)	(-)	(-)	(-)

### Approach B: Histological Findings (Table 1)

**Figure 5:**
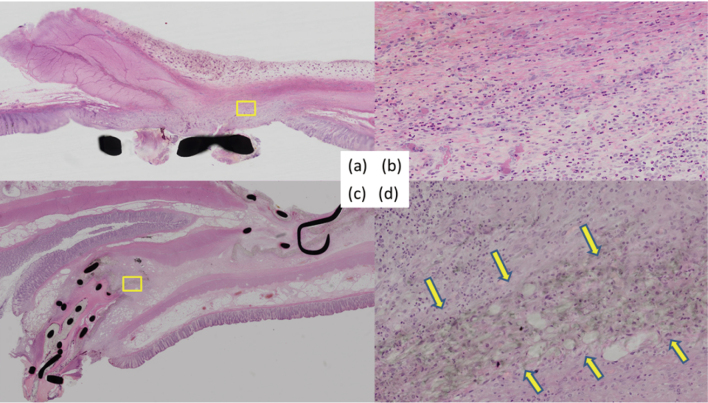
Histological findings of anastomosis (Approach B) (a) Histological findings (hematoxylin and eosin stain). (b) Anastomosis of the control case. (c) Anastomosis with the PGA sheet, with obvious remaining PGA material. (d) The PGA sheet is represented as the space between the arrows.

There was no histological leakage in the anastomosis. Histological findings showed increased fibroblasts and neutrophils in both the PGA and the control groups. Slightly more granulation formation was noted around the anastomosis in the PGA cases than in the control case. Also, in the PGA group, the PGA sheet was observed to have remained in the anastomosis. These two groups showed no other significant differences except that of the remaining the PGA sheet fibers (Figure 5). In addition, abscess formation was found around the anastomosis in one of the PGA cases. However, there was some distance between the abscess formation and PGA sheet. Moreover, no necrotic tissue was observed near the anastomosis.

## Discussion

Recently, several studies have reported the effectiveness of staple line reinforcing material for the prevention of anastomosis complications. This study assessed the usefulness and safety of a newly available PGA sheet by two approaches in animal models to show the potential of this new PGA sheet for the DST anastomosis of the colorectal surgery. Approach A was aimed to assess its utility for the reinforcement of the circular anastomosis. Approach B was aimed at addressing its safety for use in colorectal anastomosis.

In approach A, the aim was to examine the utility by measurement of RIP. Results showed not statistical differences, although the mean RIP of the PGA group was higher than that of the nonPGA group. This finding showed that the PGA sheet might reduce the AL in colorectal surgery. Naito et al reported that the high rates of complete staple formation were important for reducing anastomosis failure^16)^. They reported that reinforcement with the PGA sheet results in higher rates of complete staple formation in the approach of linear-staple on linear-staple site. In this animal model, there might be the same reinforcement with the PGA sheet in circular-staple on linear-staple site.

In approach B, laparoscopic AR was performed to clinically and histologically assess the safety of short-term outcomes of DST anastomosis postoperatively. This evaluation showed that the PGA sheet may be safe for use in colorectal surgery. Of the three pigs examined, none was found to have complications from the anastomosis. Histologic findings showed the PGA sheet remained in the anastomosis and that granulation formation had occurred around the anastomosis because of using the PGA sheet because the sheet provided scaffolds for early tissue repair. Well-built barriers of inflammatory granulation developed around the site of the anastomosis. Takagi et al showed this scaffold reduced the postoperative rate of pancreatic fistula^12)^. Early enclosure of the pancreatic leak by the granulation formation may have been achieved, and the barrier formed by the abundant fibroblast infiltration in the scaffold of the PGA sheet likely prevented the postoperative pancreatic fistula.

This study has several limitations. First, the sample size was small and thus could not demonstrate statistical difference. The results of our approaches were the higher credibility because we had used the big animal like pigs. Animal studies with a larger sample size are too difficult to continue ethically. Our results suggested that this approach was risk-free. In the future, we plan to conduct a study that confirms the PGA sheet’s effectiveness in humans. The RIP was dependent on the resting pressure of the anus; hence, the resting pressure of the anus should be measured to obtain more accurate data. Multiple RIP measurements should be made for reproducibility. However, in our study, we measured the RIP only at the point when the injected air had just overflowed. The anastomosis was broken only during one measurement, and we had only one chance of measurement per anastomosis. The duration of follow-up was short (1 week), whereas ALs may be detected anywhere from 3 to 45 days postoperatively^17, 18)^. In addition, there appears to be two peaks of when the diagnosis is made of anastomotic leaks. When leakages observed clinically, the median postoperative time of diagnosis is 7 days; when leaks are diagnosed radiographically, the median postoperative time is 16 days^8)^. In this approach, anastomotic leaks were assessed by only clinical findings. None of the pigs had clinical complications associated with anastomosis failure; therefore, the authors posited that short-term outcomes of AR might be acceptable. The other key histological finding was the abscess formation in one pig in approach B. In approach B, the PGA sheet was set intrarectally for attaching it to the mucosal surface, not within the tissue. Although there was no PGA sheet in the abscess, this finding showed that the PGA was able to lead to surgical site infection. Few studies have reported that the PGA sheet was attached to the mucosal surface^19)^. However, they did not report the histological findings. To our knowledge, our report is rare in that the PGA sheet in our study was attached to the mucosal surface of the anastomotic site of the circular stapler and assessed on the basis of histological findings. The relationship between with the clinical findings and abscess formation was unclear and must be verified. Finally, the locations of the PGA sheet in these two approaches were different. In approach A, the method to use the PGA sheet was the simplest, in which it was sandwiched between the oral and the anal side. In approach B, we considered using two PGA sheets, similar to the more clinical Endo GIA Reinforced Reload with Tri-Staple Technology (Figure 3(f)). However, our adaptation of circular Stapler with PGA sheet made it easy to slip blindly from the anus to the stump through the rectum and increased the risk of obstruction and stenosis. More PGA sheets allowed for better reinforcement of anastomosis by providing more scaffolds for early tissue repair. However, an excessive number of PGA sheets could increase the risk of obstruction and stenosis. Therefore, we used one PGA sheet, as shown in Figure 3(e). We planned to create a new device model that uses two PGA sheets for both the circular stapler and anvil. If we use a PGA sheet for the circular stapler, the PGA sheet might shift during circular stapler insertion in the anus. We thought that the PGA sheet processing was required to solve this problem. Therefore, we used the PGA sheet only in the anvil in approach B. In the future, two PGA sheets can be used more safely if a new device that employs the combination of a circular stapler and PGA sheet is developed.

In conclusion, this study experimentally assessed the application of a bioabsorbable PGA sheet in colorectal DST anastomosis clinically and histologically. Use of this sheet facilitated the setting to anvils because it was added outside the abdominal space. This study also showed the possibility that anastomosis failure may be reduced in human colorectal surgery. It is the belief of these authors that this safe device may be a valuable tool for colorectal anastomosis.

## Funding

No funding was received.

## Author contributions

Study concept and design, S Kawano, K Sakamoto; data acquisition, Y Tsuchiya, R Tsukamoto, Y Kojima; Data analysis and interpretation: S Motegi, K Kure K Sugimoto; Drafting of manuscript: S Kawano; Critical revision: M Takahashi, Atsushi Okuzawa, Kazuhiro Sakamoto; All authors read and approved the final manuscript.

## Conflicts of interest statement

The authors declare that they have no conflict of interest.
